# Black eye without a blow: eyelid ecchymosis triggered by coughing

**DOI:** 10.1093/omcr/omaf229

**Published:** 2025-11-26

**Authors:** Osamu Matsuno

**Affiliations:** Department of Allergic and Rheumatoid Disease, Osaka Habikino Medical Center, 3-7-1 Habikino, Habikino City, Osaka 583-8588, Japan

**Keywords:** cough, eyelid ecchymosis, Valsalva maneuver, cough-induced hemorrhage

**Figure 1:**
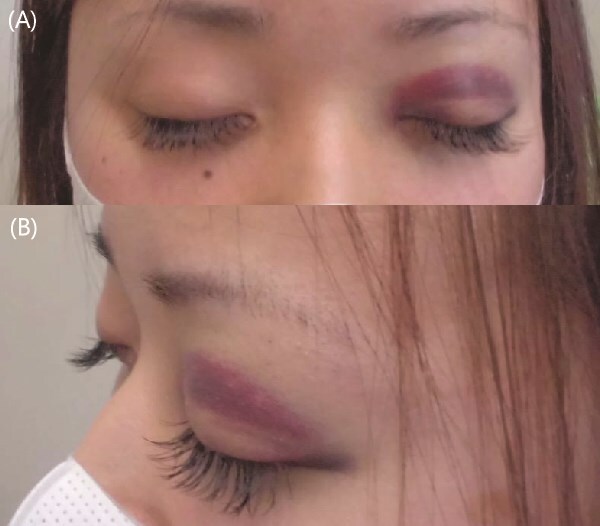
Upper (A): Frontal view showing well-demarcated purplish ecchymosis of the left upper eyelid. Lower (B): Lateral view highlighting the contour and extent of the lesion.

A 27-year-old woman developed sudden ecchymosis of the left upper eyelid immediately after a paroxysm of forceful coughing (Fig. 1). She had experienced a persistent dry cough for three weeks following a febrile illness (maximum temperature, 38.7°C) accompanied by sore throat. She denied any history of trauma, eye rubbing, bleeding disorders, or use of anticoagulant medications. When there is a clear precipitating factor such as forceful coughing, and more common or serious conditions (e.g., bleeding disorders) have been appropriately excluded, consideration of rare benign etiologies can prevent unnecessary investigations or misdiagnosis. Notably, the patient had no history of over-the-counter NSAID use, nor any family history of bleeding disorders.

Physical examination revealed a well-demarcated purplish ecchymosis localized to the left upper eyelid. There was no evidence of subconjunctival hemorrhage, swelling, or tenderness. Her vital signs were stable, and she appeared systemically well. Chest auscultation, chest radiography, pulmonary function tests, and laboratory investigations—including complete blood count and coagulation profile—were all within normal limits.

A benign, self-limited rupture of periorbital capillaries due to transient venous pressure elevation during coughing—consistent with a Valsalva-induced hemorrhage—was diagnosed. The ecchymosis resolved spontaneously within one week without any intervention.

Although periorbital ecchymosis (commonly known as ‘raccoon eyes’ or ‘panda sign’) is typically associated with basal skull fractures, it has also been reported in systemic conditions such as amyloidosis and lymphoma, or after episodes of sneezing or vomiting [1, 2]. However, isolated unilateral eyelid ecchymosis following coughing, without subconjunctival involvement or underlying systemic disease, is exceedingly rare. A similar case involving bilateral periorbital ecchymosis in a child was recently published in the *New England Journal of Medicine*; however, our case is unique due to its adult onset, unilateral eyelid involvement, absence of subconjunctival hemorrhage, and complete spontaneous resolution without any intervention [3].

This case highlights the importance of recognizing this rare but benign clinical presentation, which may be mistaken for trauma or hematologic disorders, potentially resulting in unnecessary investigations or misdiagnosis.
